# Are we missing lifetime COPD diagnosis among people with COPD recorded death? A population-based retrospective cohort study

**DOI:** 10.3399/BJGPO.2022.0060

**Published:** 2022-09-21

**Authors:** Alicia Gayle, Alexandra Lenoir, Cosetta Minelli, Jennifer Quint

**Affiliations:** 1 National Heart and Lung Institute, Imperial College London, London, UK; 2 National Institute for Health Research, Imperial Biomedical Research Center, London, UK; 3 Epidemiology Department, AstraZeneca, Cambridge, UK; 4 Department of Respiratory Medicine, Centre Hospitalier Universitaire Vaudois, Lausanne, Switzerland; 5 Gesundheitsamt Fürstenfeldbruck, Fürstenfeldbruck, Germany

**Keywords:** pulmonary disease, chronic obstructive, diagnosis, general practice, primary health care, Clinical Practice Research Datalink

## Abstract

**Background:**

The British Lung Foundation (BLF) has previously estimated that there are 2.2 million people in the UK who have symptoms, but no diagnosis, of chronic obstructive pulmonary disease (COPD).

**Aim:**

To investigate the proportion of patients with a missed COPD diagnosis among those with COPD as the cause of death on their death certificate, and how this has changed over a period of 17 years (2000–2017).

**Design & setting:**

Clinical Practice Research Datalink (CPRD) Aurum and GOLD primary care data were linked with Office for National Statistics (ONS) mortality data and Hospital Episode Statistics (HES) data. Adults who died between 2000 and 2017 with COPD as their main cause of death were included.

**Method:**

Using a range of diagnostic COPD criteria, the proportion of patients with a missed COPD diagnosis was estimated, and the demographic and clinical characteristics of patients with and without prior COPD diagnosis were described, using a mixed-effect logistic regression model.

**Results:**

Depending on the COPD definition used, between 96% and 27% of the 78 621 patients included received a diagnosis of COPD before death. Using presence of a COPD Read or SNOMED CT code and performed spirometry as a main definition, just over half of the patients (52%) had received a COPD diagnosis overall, with a proportion of those who did not decreasing from 91% in 2000 to 31% in 2017 (*P_trend_* <0.001).

**Conclusion:**

The proportion of people with COPD-recorded death and who had received a diagnosis of COPD has improved (increased) over time, and currently represents the majority of them. This suggests that few patients are now being missed.

## How this fits in

COPD is often diagnosed when patients are at a severe stage and, in some cases, is completely missed in patients’ medical history until a terminal event. This is the largest population-based study linking COPD mortality with COPD diagnosis in general practice. It demonstrates that over the past decades the proportion of people with COPD as the cause of death but *without* a COPD diagnosis in their medical record has decreased. In the past decade, expansion of targeted screening programmes, better disease awareness, case-finding, and diagnostic services may partly explain the narrowing gap. Ensuring that granular information on patients' clinical status is recorded in medical records is of high importance as it has implications for the quality of care delivered.

## Introduction

The BLF estimated in 2007 that there were 2.2 million symptomatic but undiagnosed people with COPD in the UK.^1^ Opportunities for diagnosis are missed in 85% of patients up to 5 years before diagnosis.^2^ People sometimes have mild or infrequent symptoms, making it difficult to be diagnosed. Clinicians may attribute respiratory symptoms to other diseases where symptoms overlap, including heart failure, asthma, and other chronic lung conditions. Another potential issue may be that spirometry, the essential test to detect airway obstruction, may not be available or is misinterpreted due to lack of recognition of poor quality, or use of inappropriate reference values.^3^

National Institute for Health and Care Excellence (NICE) guidelines (NG115) recommend that diagnosis of COPD is established based on confirmation of post-bronchodilator airflow obstruction forced expiratory volume (FEV)_1_/forced vital capacity (FVC) <0.7.^4^ While spirometry can be performed in primary care, diagnostics are often conducted in secondary care settings where results may or may not be shared with GPs, or when shared may not be coded and entered into the medical record in a way that is easily accessible to clinicians or researchers. Quint *et al* previously validated COPD diagnosis in primary care data, concluding that using COPD codes alone (positive pressure ventilation [PPV] 86.5%; 77.5%–92.3%) or in combination with recorded spirometry and prescription of specific COPD medications (PPV 89.4%; 80.7%–94.5%) accurately identify the majority of patients with COPD in medical records.^5^

The present study measures the proportion of patients with a diagnosis of COPD before death (assuming a COPD-recorded death is the gold standard), using a range of diagnostic definitions found to have high sensitivity and specificity in primary care data (Box 1).

Box 1 **Chronic obstructive pulmonary disease (COPD) definitions**

**Table:** 

Group	Definition
A	Specific COPD code only:Diagnostic COPD code or record of acute exacerbation of COPD in either primary care or secondary care data using previously validated Read or SNOMED-CT or International Classification of Diseases, Tenth Revision (ICD-10) codes^11^ (see Supplementary Tables S1 and S2 for additional codes used to identify COPD and COPD exacerbations using SNOMED-CT codes)
B	Specific COPD code and record of spirometry:Record of spirometry being performed at any time in a patient's medical history
C	Specific COPD code and medication prescribed within 4 weeks of a diagnostic code
D	Specific COPD code, presence of spirometry and medication prescribed within 4 weeks of a diagnostic code
E	Specific COPD code with post-bronchodilator spirometry confirmed obstruction(forced expiratory volume [FEV]_1_/forced vital capacity [FVC] <0.7)
F	Specific COPD code with post-bronchodilator spirometry confirmed obstructionFEV_1_/FVC < lower limit of normal (LLN)

## Method

### Data source

This cohort study utilised data provided by the CPRD, which include routinely collected primary care data from 1073 GP practices across the UK and information for approximately 24 million patients. It has been shown to be representative of the national demographic, including age and sex.^6,7^ Data in CPRD contain information on clinical diagnoses, healthcare consultations, prescribed medications by primary care providers (PCPs), laboratory tests, and referrals to medical specialists. Linked socioeconomic data from the Index of Multiple Deprivation (IMD), and secondary care data spanning accident and emergency (A&E) visits and admissions from HES were provided for this study by CPRD. Approximately 75% of CPRD practices in England are eligible for linkage. Primary care data from practices using two general practice software systems were combined: CPRD GOLD, which includes general practices using Vision software, and Aurum, which includes practices using EMIS software^7^. Patients who belonged to practices that migrated from Vision to EMIS software (GOLD to Aurum) were removed from the GOLD data set to avoid duplication.^6,8,9^ Linked pseudonymised mortality data from ONS, socioeconomic data from the IMD, and secondary care data from HES were provided for this study by CPRD for patients in England (May 2019, linkage set 17).

### Patient population

**Figure 1.**
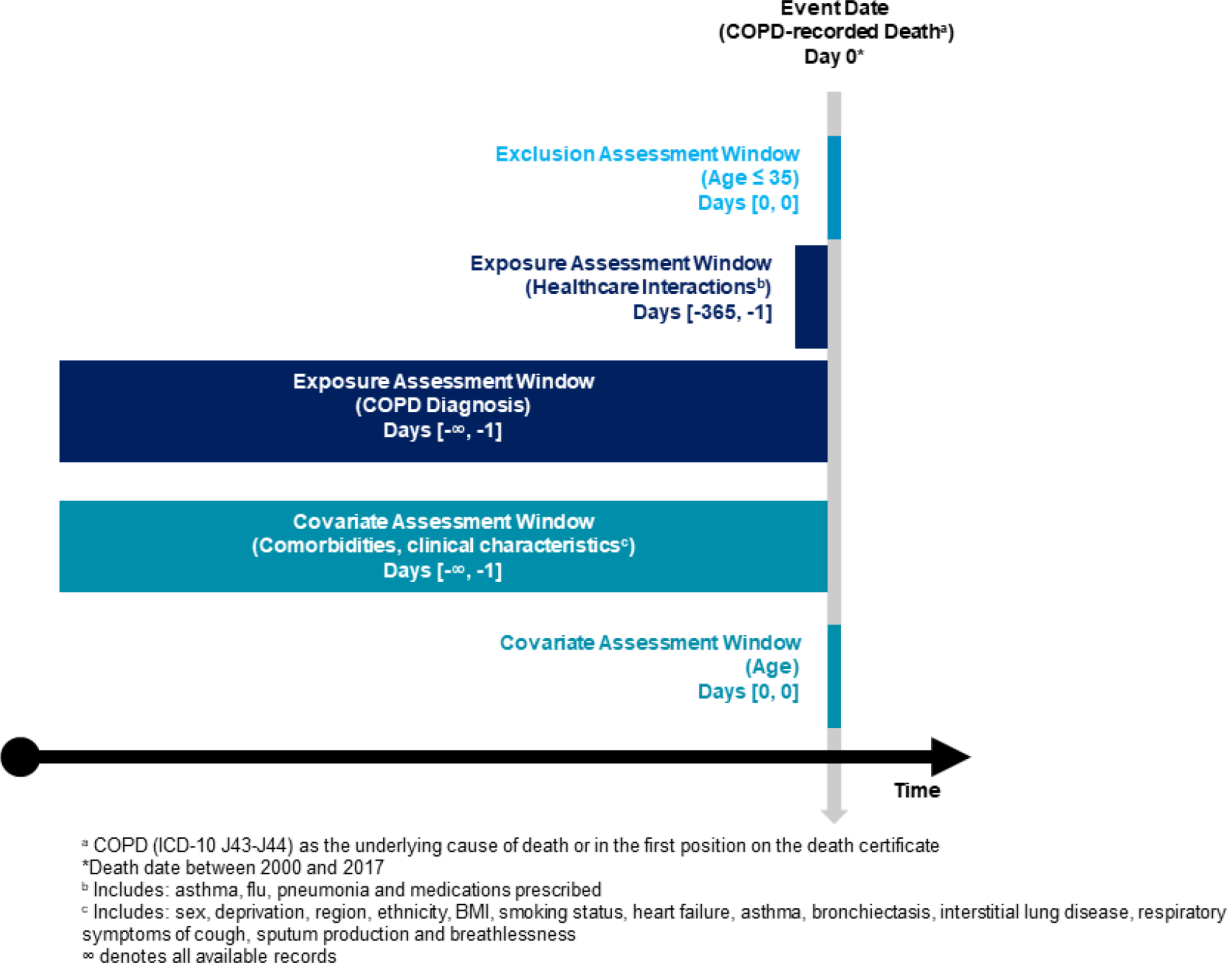
Study design. BMI = body mass index. COPD = chronic obstructive pulmonary disease. ICD-10 = International Classification of Diseases, Tenth Revision.

All patients were included who died in England between 2000 and 2017 whose underlying cause of death or first position cause of death on the death certificate was COPD (International Classification of Diseases, Tenth Revision [ICD-10] codes J43–J44), as all patients experiencing a death owing to COPD should have had a prior diagnosis. Patients who were registered at their GP practice for at least 1 year before death were included. Patients were excluded if they were aged ≤35 years at the time of death (Figure 1).

The study retrospectively looked for evidence of COPD diagnosis based on interpretations of the NICE diagnostic guidelines^4^ and previously validated CPRD diagnostic algorithms.^5^ The definitions used to diagnose COPD are presented in Box 1 and range from loose definitions to the strictest.

### Statistical analysis

To illustrate the impact of choice of diagnostic criteria, and for ease of comparison, the loosest criteria (A: COPD code only) was compared with the most strict (E: COPD code and confirmation of airflow obstruction). Definition B was then considered (COPD code and presence of spirometry), which was the main definition for the purposes of further analysis, as this was the most robust definition according to NICE guidelines.^4^

Variation over time in the proportion of patients who received a COPD diagnosis was evaluated using the Cochrane–Armitage test for trend. Differences in the specified covariates between those with and without a COPD diagnosis were tested using a Wilcoxon rank sum test for continuous variables (as they were not normally distributed), and a χ^2^ test for binary and categorical variables inclusive of missing categories. Factors associated with missed COPD diagnosis were assessed using a two-level mixed-effect logistic regression model with random intercept by GP practice to account for the multi-level nature of the data. The model was adjusted for age (year of birth), sex, smoking status (currently smokes, formerly smoked, never smoked, or unknown), body mass index ([BMI] underweight, normal weight, overweight, obese, or unknown), year of death, IMD, ethnic group, and comorbid respiratory diseases (asthma ever, interstitial lung disease [ILD], or bronchiectasis).

As a sensitivity analysis, the analysis was repeated after excluding patients with any recorded asthma diagnosis, as the overlap between COPD and asthma^10^ may have biased the proportion of patients who do not receive a diagnosis of COPD before death; previous research has shown that a large proportion of diagnosed patients with asthma later have COPD listed as their underlying cause of death.^11^

## Results

**Figure 2.**
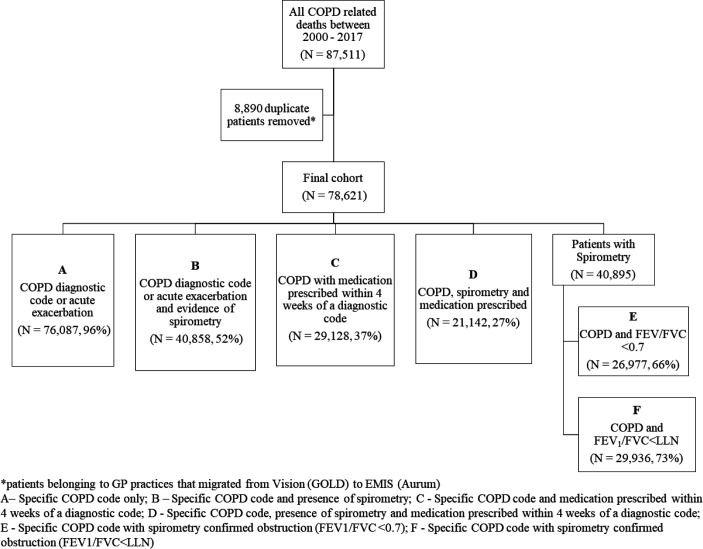
Proportion of patients with evidence of chronic obsructive pulmonary disease (COPD) by diagnostic criteria. FEV/FVC = forced expiratory volume/forced vital capacity. LLN = lower limit of normal

A total of 87 511 patients were included who had COPD as the main cause of death. Among them, 18 945 patients belonged to practices included in GOLD, and 59 676 in Aurum resulting in a cohort of 78 621 patients (after removing duplicates). Patients had a median of 14.6 years of follow-up before death (interquartile range [IQR]: 7.5–24.5). Depending on the definition of COPD used (Box 1), it was estimated that as many as 96% or as few as 27% of patients had evidence of a diagnosis of COPD in their medical records (Figure 2). Just over half of patients (52%, n = 40 895) had evidence of spirometry in their medical records. When considering diagnostic criteria, including evidence of airflow obstruction, the proportion of patients with evidence of COPD was estimated between 66% using definition E and 73% using definition F (Figure 2).

**Figure 3.**
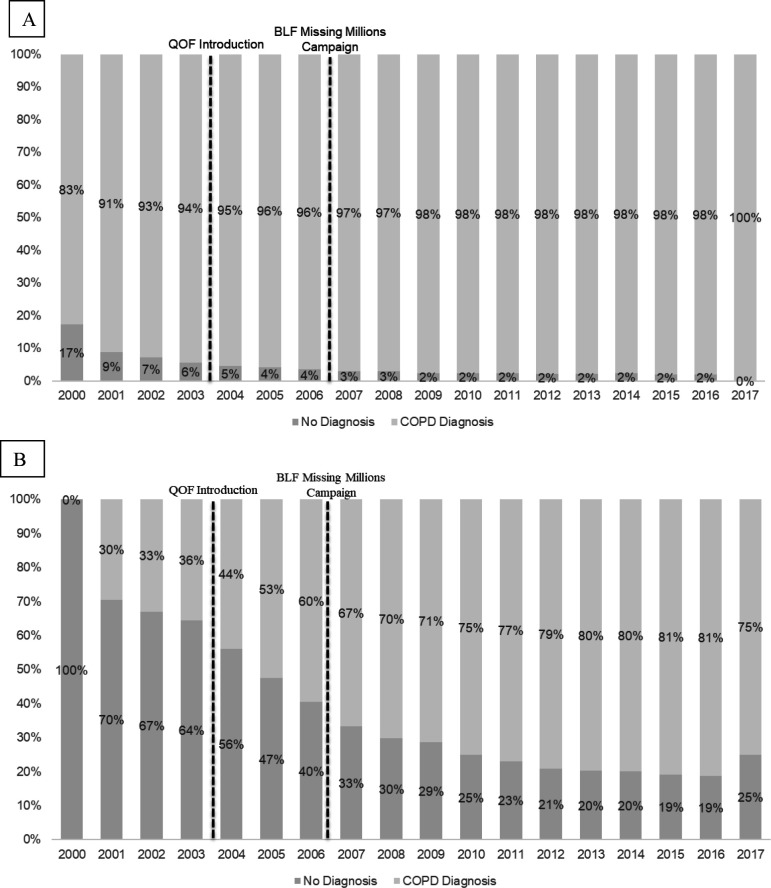
Proportion of patients with a missed COPD diagnosis by year of death (panel A shows definition A, panel B shows definition E). BLF = British Lung Foundation. COPD = chronic obstructive pulmonary disease. QOF = Quality and Outcomes Framework.

Comparing the loosest criteria (A: COPD code only) with the most strict (E: COPD code and confirmation of airflow obstruction), the proportion of patients with missed COPD diagnosis decreased over the study period, from 17% in 2000 to 0% in 2017 (*P_trend_* <0.001) using definition A (Figure 3), and from 100% in 2000 to 25% in 2017 (*P_trend_* <0.001) using definition E (Figure 3). Supplementary Table S3 describes patient characteristics between those with and without a diagnosis, according to definitions A and E.

Using the main definition (B: COPD code and presence of spirometry), just over half (*n* = 40 858, 52%) had evidence of COPD diagnosis in their medical records, and the proportion of patients with missed COPD diagnosis decreased over the study period from 91% in 2000 to 31% in 2017 (p trend <o.oo1). Table 1 describes patient characteristics comparing those with and without a COPD diagnosis in their lifetime, using this definition. Among the 37 763 (48%) patients with a missed COPD diagnosis, under half had an asthma diagnosis at any point in their medical history (*n* = 15 645, 41%), and a large proportion had reported feeling breathless at least once in their medical history (*n* = 27 351, 72%). Among those with evidence of COPD diagnosis, the prevalence of asthma was higher (*n* = 24 846; 61%), and the majority reported feeling breathless (*n* = 38 179, 93%).

**Table 1.** Characteristics of patients with missed COPD diagnosis compared with those with diagnosed COPD using the main definition (B: COPD code and presence of spirometry)

		Missed COPD diagnosis	COPD diagnosis	*P* value
		*n* = 37 763	*n* = 40 858	
Age at death		80 (73–87)	79 (72–85)	<0.001
Sex	Female	18 339 (48.6%)	18 907 (46.3%)	<0.001
IMD	1 (least deprived)	5544 (14.7%)	5812 (14.2%)	0.085
	2	6508 (17.2%)	7221 (17.7%)	
	3	7258 (19.2%)	7965 (19.5%)	
	4	8205 (21.7%)	9088 (22.2%)	
	5 (most deprived)	9986 (26.4%)	10 693 (26.2%)	
	Missing	262 (0.7%)	79 (0.2%)	
Region	South of England	20 154 (53.4%)	20 836 (51.0%)	<0.001
	North of England	17 609 (46.6%)	20 022 (49.0%)	
Ethnic group	White	31 593 (83.7%)	37 949 (92.9%)	<0.001
	Asian	236 (0.6%)	223 (0.5%)	
	Black	162 (0.4%)	121 (0.3%)	
	Mixed or other	253 (0.7%)	200 (0.5%)	
	Missing	5519 (14.6%)	2365 (5.8%)	
BMI (kg/m^2^)	Underweight (<18.5)	3004 (8.0%)	6433 (15.7%)	<0.001
	Healthy weight (18.5–24.9)	11 697 (31.0%)	17 361 (42.5%)	
	Overweight (25.0–39.9)	5727 (15.2%)	8274 (20.3%)	
	Obese (>40)	3399 (9.0%)	5619 (13.8%)	
	Missing	13 936 (36.9%)	3171 (7.8%)	
Comorbid conditions	Heart failure	14 716 (39.0%)	17 001 (41.6%)	<0.001
	Asthma (ever)	15 645 (41.4%)	24 846 (60.8%)	<0.001
	Asthma (year before)	3177 (8.4%)	4318 (10.6%)	<0.001
	Bronchiectasis	2324 (6.2%)	4872 (11.9%)	<0.001
	Interstitial lung disease	1686 (4.5%)	2795 (6.8%)	<0.001
Symptom burden	Cough symptoms	13 453 (35.6%)	27 843 (68.1%)	<0.001
	Sputum production	6752 (17.9%)	18 886 (46.2%)	<0.001
	Breathlessness	27 351 (72.4%)	38 179 (93.4%)	<0.001
Smoking status	Currently smokes	13 201 (35.0%)	13 072 (32.0%)	<0.001
	Formerly smoked	9478 (25.1%)	23 366 (57.2%)	
	Never smoked	5061 (13.4%)	3410 (8.3%)	
	Missing smoking status	10 023 (26.5%)	1010 (2.5%)	

BMI = body mass index. COPD = chronic obstructive pulmonary disease. IMD = Index of Multiple Deprivation. Data are presented as *n* (%) for categorical measures, and median and interquartile range for numerical variables. Comorbidities assessed as occurring at any point in medical record before death.

A number of factors were identified as being independently associated with missed diagnosis in the multiple regression model (Table 2). Missed COPD diagnosis was more likely in individuals who had no recorded smoking status compared with a ‘currently smokes’status. Similarly, never having smoked, being overweight or obese, and being in Black, Asian, or unknown ethnic groups were associated with increased odds of missed diagnosis. Conversely, a number of factors reduced the likelihood of missed diagnosis; for example, calendar time, formerly smoking compared with current smoking, being underweight, region, and comorbid asthma, bronchiectasis, and ILD.

**Table 2.** Adjusted odds ratios for missed COPD diagnosis for all factors tested in the multiple regression model

		Odds ratio (95% CI)	*P* value
Birth year		0.99 (0.99 to 1.00)	<0.001
Female		1.16 (1.12 to 1.21)	<0.001
Year of death		0.86 (0.86 to 0.87)	<0.001
Smoking Status	Formerly Smoked vs Currently Smokes	0.42 (0.40 to 0.43)*	<0.001
	No Smoking Record vs Currently Smokes	4.52 (4.13 to 4.95)*	<0.001
	Never smoked vs Currently smokes	1.26 (1.18 to 1.35)*	<0.001
BMI (kg/m^2^)	Underweight vs Healthy Weight	0.63 (0.59 to 0.67)*	<0.001
	Overweight vs Healthy Weight	1.15 (1.10 to 1.21)*	<0.001
	Obese vs Healthy Weight	1.11 (1.05 to 1.17)*	<0.001
Region	North of England vs South of England	0.86 (0.80 to 0.92)*	<0.001
IMD Quintile	2 vs 1 (least deprived)	0.96 (0.90 to 1.03)	0.23
	3 vs 1	0.96 (0.90 to 1.03)	0.255
	4 vs 1	0.95 (0.88 to 1.02)	0.122
	5 vs 1	1.02 (0.95 to 1.10)	0.63
Ethnic group	Black vs White	1.87 (1.37 to 2.54)*	<0.001
	Mixed/Other vs White	1.08 (0.83 to 1.39)	0.654
	Asian vs White	1.54 (1.21 to 1.96)*	<0.001
	Unknown vs White	1.28 (1.20 to 1.38)*	<0.001
Comorbid conditions	Asthma	0.39 (0.38 to 0.41)*	<0.001
	Interstitial lung disease	0.92 (0.85 to 1.00)*	0.04
	Bronchiectasis	0.72 (0.68 to 0.77)*	<0.001

IMD = Index of Multiple Deprivation. Northern regions include North East, North West, Yorkshire and the Humber, East Midlands, West Midlands.Southern regions include East of England, South West, South Central, London, South East Coast.

Statistically significant results are indicated in **bold** with asterisk.

Findings from the sensitivity analysis removing patients with asthma were consistent with those of the main analysis; missed COPD diagnosis remained more likely in individuals with no recorded smoking status compared with those who currently smoke (OR 5.34; 95% CI = 4.57 to 6.23), and less likely for former compared with current smoking (OR 0.39; 95% CI = 0.37 to 0.41), presence of bronchiectasis (OR 0.68; 95% CI = 0.62 to 0.76), and calendar time (year of death, OR 0.90; 95% CI = 0.98 to 0.99) (see Supplementary Table S4).

## Discussion

### Summary

This population-based retrospective cohort study estimated the proportion of people with death attributed to COPD who had evidence of a COPD diagnosis in their medical records. Regardless of the definition used, the proportion of patients with a missed diagnosis during their lifetime decreased between 2000 and 2017, suggesting that there were improvements in COPD detection and/or recording. However, the proportion of people missed varied widely depending on the definition of COPD used; anywhere from 96% to as low as 27% of patients were identified with diagnosed COPD depending on the definition used, with those definitions which included recorded spirometry having lower proportions. Patients without evidence of a COPD diagnosis were more likely to have no smoking information recorded, little or no respiratory symptoms, and fewer comorbid conditions, suggesting less frequent contact with health care and therefore reduced opportunity for diagnosis.

### Comparison with existing literature

Globally, the estimated proportion of undiagnosed patients with COPD varies.^12^ In a Danish prospective cohort study, Çolak *et al* concluded that 78% of people at high risk of developing COPD (former and current smokers aged ≥40 years with a cumulated tobacco consumption of 10 pack-years or higher, and who did not have asthma) had undiagnosed disease.^13^ Conversely, among a random sample of adults without a previous history of asthma or COPD in Canada, Preteroti *et al* estimated that 12% of adults with respiratory symptoms had spirometry-confirmed COPD.^14^ Miravitlles *et al* investigated the prevalence of persistent airflow limitation in Spain using a definition of post-bronchodilator FEV_1_/FVC <0.7; among 4274 adults aged 40–80 years, the prevalence of persistent airflow obstruction was 10%, with only 27% of those subjects identified having previously received a diagnosis of COPD.^15^ Using data obtained from the National Health and Nutrition Examination Survey (NHANES) in the US, Hangaard *et al* estimated that among 1098 subjects with post-bronchodilator FEV_1_/FVC <0.7 or LLN, 92% had no previous diagnosis of COPD, defined by patient self-report.^16^ The current estimate of diagnosed COPD differs from previous research owing to the definition of the study population: while the previous research in this area identifies patients prospectively from mid-age or select a group of high-risk individuals whom guidelines recommend should receive a diagnosis, the present study identified COPD diagnosis retrospectively and considered all available medical records to detect physician-identified disease.

Differences between those who received a COPD diagnosis and those who were missed may be further explained by factors that were not possible to measure. While the majority of diagnosed patients had COPD recorded in both primary care and secondary care, the communication between these settings is not always complete. Practice-related reasons are important for missing COPD diagnoses, staff trained to carry out spirometry correctly according to validated guidelines are sometimes short in supply, and the procedure may not be possible through virtual healthcare delivery.^17^ A recent BLF report estimated that 46 000 people had a missed diagnosis during the pandemic, with 34% of those with COPD not receiving spirometry at diagnosis.^18^ In the present study, a small proportion of patients without recorded diagnosis while alive had spirometry performed, and where this was done, the majority did not have lung function measures that met diagnostic criteria for COPD. Active case-finding can identify around 70% more cases than opportunistic examination,^19^ and significant variation in this rate has been shown around the country.^20^ Many of the non-diagnosed patients presented with no or few respiratory symptoms, which may prevent a GP investigating possible COPD, therefore a targeted screening-based approach would be needed to estimate the proportion of less symptomatic patients with COPD.

Improvements were observed in the proportion of patients with a diagnosis over time, which may be partially attributed to improvements in recording of data. When considering evidence of COPD diagnosis defined by presence of a diagnostic code only (A), the proportion of patients missed reduced to 0% in 2017, demonstrating that diagnostic coding of COPD is becoming more complete among those with a COPD-recorded death. The Quality and Outcomes Framework (QOF) was introduced in 2004 and provided incentives for primary care practices to keep registers of patients with chronic disease;^21^ practices were rewarded for documenting confirmed diagnosis of COPD with spirometry, recording FEV_1_, checking inhaler technique, offering flu vaccination, and recording smoking status of all patients identified on the registers. This may have contributed towards the 50% increase over a 10-year period in the recorded prevalence of COPD between 2000 and 2009,^22^ as well as the declines in the proportion of patients who had a missed diagnosis after 2004 (Figure 3). Improvements in COPD diagnosis may also be attributed to increased case-finding and targeted screening initiatives at the local and regional level.^22,23^ The BLF’s *Missing Millions* campaign raised awareness of the possibility that a large proportion of patients with COPD were undiagnosed.^24^

### Strengths and limitations

A major strength of this study was the breadth of data available: linked data between primary and secondary care provide near complete records of the patient clinical journey. While outpatient, results of spirometry performed in hospital and hospital-prescribing information was not included in this study, it is expected the impact of this to be minimal, as outpatient activity is poorly coded and the majority of diagnostic services sit within primary care. Diagnosis of COPD was based on physician coding, which relies on complete data entry and does not include free-text notes; however, this previously validated definition using a combination of presence of a COPD diagnostic code with evidence of spirometry in the medical record has been shown to have high positive predictive value.^5^

This is the first study to use a COPD-recorded death as the reference for confirmed diagnosed COPD, and this approach assumes that death certificates are a clinical 'gold standard'. Mortality data for England and Wales are nearly 100% complete, a large proportion of registered deaths are certified by a medical practitioner, and accuracy of recording has improved over time.^25^ However, a number of limitations of this study design apply. The authors were not able to query death certificates to validate the COPD cause of death. Additionally there may be differences in cause of death certification between those dying in hospital compared with those dying in other settings, where access to diagnostic measures are less readily available. Further, undiagnosed patients with COPD may have died due to other causes and therefore this study does not reflect a complete assessment of the number of patients who are missed. Further research inclusive of broader causes of death is needed for a complete estimate of these patients.

### Implications for research and practice

The majority of patients with COPD received a diagnosis before a COPD-related death. Those who did not were more likely to have never smoked and did not present with symptoms to the GP, and therefore may not have been flagged to have diagnostic spirometry, suggesting that there is room for improvement in the recording of spirometry. Obtaining granular information on patients' clinical status in medical records is of high importance as it has implications on the quality of care delivered. Disease management plans are informed by the details included in patient records, regardless of where a patient is managed; therefore, there is a need to ensure that these vital tests are performed, recorded, and shared transparently between primary and secondary care. However, the authors acknowledge that clinical coding of COPD in the present study was near complete for all patients, implying that the majority of patients would be included in disease registers.

## Data Availability

Data were linked by NHS Digital, the statutory trusted third party for linking data, using identifiable data held only by NHS Digital. Select general practices consent to this practice level with individual patients having the right to opt out. Use of HES and ONS data is Copyright ^©^ (2021), re-used with the permission of The Health & Social Care Information Centre, all rights reserved. Data are available on request from the CPRD. Their provision requires the purchase of a license, and this license does not permit the authors to make them publicly available. This work used data from the version collected in May 2019 and have clearly specified the data selected each Methods section. To allow identical data to be obtained by others, via the purchase of a license, the code lists will be provided upon request. Licenses are available from the CPRD (http://www.cprd.com" http://www.cprd.com): The Clinical Practice Research Datalink Group, The Medicines and Healthcare products Regulatory Agency, 10 South Colonnade, Canary Wharf, London E14 4PU.

## Data

Data were linked by NHS Digital, the statutory trusted third party for linking data, using identifiable data held only by NHS Digital. Select general practices consent to this practice level with individual patients having the right to opt out. Use of HES and ONS data is Copyright © (2021), re-used with the permission of The Health & Social Care Information Centre, all rights reserved. Data are available on request from the CPRD. Their provision requires the purchase of a license, and this license does not permit the authors to make them publicly available. This work used data from the version collected in May 2019 and have clearly specified the data selected each Methods section. To allow identical data to be obtained by others, via the purchase of a license, the code lists will be provided upon request. Licenses are available from the CPRD (http://www.cprd.com): The Clinical Practice Research Datalink Group, The Medicines and Healthcare products Regulatory Agency, 10 South Colonnade, Canary Wharf, London E14 4PU.
